# Efficacy and Safety of Rivaroxaban versus Warfarin for the Treatment of Acute Pulmonary Embolism: A Real-World Study

**DOI:** 10.1155/2020/6813492

**Published:** 2020-06-26

**Authors:** Yan Huang, Linli Duan, Wenjun He, Cheng Hong, Yehui Guo, Xinni Wang, Nuofu Zhang, Yanghang Chen, Tao Wang, Jian Wang, Chunli Liu

**Affiliations:** ^1^State Key Laboratory of Respiratory Disease, National Clinical Research Center for Respiratory Disease, Guangzhou Institute of Respiratory Health, The First Affiliated Hospital of Guangzhou Medical University, Guangzhou, Guangdong 510120, China; ^2^Xishuangbanna Dai Autonomous Prefecture People's Hospital, Xishuangbanna Dai Autonomous, Yunnan, China; ^3^The Second Affiliated Hospital of Guangzhou Medical University, Guangzhou, Guangdong, China

## Abstract

**Background:**

Pulmonary embolism (PE) is a life-threatening disease. Target-specific anticoagulant rivaroxaban is a direct factor Xa inhibitor that can be safely used without laboratory monitoring.

**Objective:**

To investigate the efficacy and safety of rivaroxaban versus warfarin for the treatment of acute pulmonary thromboembolism in real-world clinical practice.

**Method:**

This was a semiretrospective, semiprospective, and real-world trial involving 128 patients with acute symptomatic pulmonary embolism with or without active tumor or frailty. We compared rivaroxaban to the standard therapy consisting of low-molecular-weight heparin combined with warfarin. The primary efficacy outcome was absorption of thrombus. The principal safety outcome was bleeding episode.

**Results:**

There was no significant difference in thrombus absorption between rivaroxaban and standard therapy after 3-month treatment (*P* = 0.798, 95% confidence interval (CI) 0.686 to 1.336) or more than 6-month treatment (*P* = 0.534, 95% confidence interval (CI) 0.795 to 1.556). There was no decline in efficacy (including computed tomographic pulmonary angiography and recurrence) when the rivaroxaban dose was reduced to 10 mg once daily after 3 months of administration. The ratio of patients without bleeding was 48.84% for rivaroxaban and 19.05% for standard therapy (*P* = 0.001). There was no significant difference in rivaroxaban monotherapy subgroups (including frail patients, tumor patients, and thrombolysis or nonthrombolysis at intermediate-high-risk patients).

**Conclusion:**

In this real-world study, the efficacy and safety of rivaroxaban alone was not different to standard therapy for pulmonary emboli absorption. With an extension in treatment duration, the rivaroxaban regimen had a higher efficacy and safety than standard therapy and there was no decline in treatment efficacy when the rivaroxaban dose was reduced to 10 mg once daily.

## 1. Introduction

Pulmonary embolism (PE) is a disease with high morbidity and mortality [1]. Because the usage of rivaroxaban is increasing in actual clinical practice, it is necessary to compare the rivaroxaban monotherapy regimen with standard therapy [2]. Even though several guidelines for the treatment of acute pulmonary embolism with rivaroxaban have been published, there are many PE patients with specific clinical characteristics including frailty (e.g., elderly, low body weight), oncology, or at intermediate-high-risk. Among them, elderly patients have a high incidence of bleeding [3], which increases when coupled with warfarin treatment; however, there are no guidelines for reducing the anticoagulant dose for that population. For low-weight patients, the efficacy of novel oral anticoagulants (NOACs) depends on its plasma concentrations, which are closely related to body mass/BMI. A previous RCT study in the field of atrial fibrillation reported that most NOACs increased the rate of bleeding in low-weight patients; therefore, the dose of apixaban and edoxaban should be adjusted according to the body weight [4]. However, there is no evidence which informs the adjustment of the dose of rivaroxaban based on the body weight. Tumors are the strongest independent risk factors of all-cause mortality in patients with venous thromboembolism (VTE) [5, 6]. Because of the recent development of NOACs, there is almost no evidence on whether rivaroxaban can be used to prevent VTE in cancer patients. For intermediate-high-risk patients, systemic thrombolysis improved the potential of hemodynamic disturbances and improved treatment outcomes concurrently [7]. However, the PEITHO study reported the rate of hemorrhagic stroke was increased by 2% after thrombolysis in intermediate-high-risk patients [8]. Furthermore, for intermediate-high-risk patients, thrombolysis can increase the efficacy and risk of bleeding, but there have been no reports on whether rivaroxaban can achieve the same effect as thrombolysis without increasing the risk of bleeding.

We previously compared rivaroxaban monotherapy with standard therapy based on gene testing to adjust the dose of warfarin [2]. The results showed that the efficacy of rivaroxaban monotherapy was better than standard therapy and had a lower incidence of bleeding. Because of the small sample size in our previous study, the current study continued the comparison of the two regimens in real-world clinical practice.

Our trial consisted of two parts: (1) comparison of the efficacy and safety of rivaroxaban and warfarin and (2) subgroup analysis of rivaroxaban, which contained (a) efficacy and safety of frail and nonfrail patients in the rivaroxaban regimen; (b) efficacy and safety of tumor and nontumor patients with pulmonary embolism; and (c) efficacy and safety of the rivaroxaban regimen and thrombolytic therapeutic regimen in intermediate-high-risk patients.

## 2. Materials and Methods

### 2.1. Patients

This study was approved by the Ethics Committee of The First Affiliated Hospital of Guangzhou Medical University (trial registration: this study has been registered at ClinicalTrials.gov: ChiCTR-TRC-14005223; 12 September 2014). Inclusion criteria were ≥18 years, acute pulmonary embolism diagnosed by computed tomographic pulmonary angiography (CTPA), and ventilation/perfusion (V/Q), with treatment of rivaroxaban monotherapy or standard-therapy. Exclusion criteria were no thrombus in chronic pulmonary embolism patients, or patients who received thrombectomy or vena cava filter.

### 2.2. Assignment and Treatment Regimens

Patients assigned to the rivaroxaban group received 15 mg twice daily for the first 3 weeks, 20 mg once daily from 3 weeks to 3 months, and followed by 10 mg once daily for long-term anticoagulation. Patients assigned to the standard-therapy group received a dose of warfarin of 3–5 mg once daily followed by low-molecular-weight heparin (LMWH) 0.1 ml per 10 kg body weight twice daily. LMWH was discontinued when the international normalized ratio (INR) was 2.0–3.0 over consecutive days, and then, INR was detected once a week. The dose of warfarin was adjusted to maintain an INR of 2.0–3.0, and the INR was detected once a month.

For rivaroxaban subgroups, frail patients included patients aged >70 years or weight < 45 kg or BMI < 18. Tumor patients were defined as patients with active tumors, including patients who were undergoing radiotherapy and chemotherapy or were postoperative. Patients in the third subgroup had thrombolysis or no thrombolysis and were at intermediate-high risk.

### 2.3. Assessments

The primary efficacy outcome was absorption of thrombus, which was detected by CTPA or V/Q scan, and graded as disappearance, effective, no effect, or progressed to worse according to the following definitions:

*Disappearance*. 100% of the thrombus was resorbed.

*Effective*. >25% of the thrombus resorbed.

*No Effect*. <25% of the thrombus resorbed

*Progressed to Worse*. pulmonary embolism progressed to worse.

The principal safety outcome was bleeding, which included anticoagulant-induced bleeding, and liver or kidney dysfunction. Bleeding was defined and graded as (1) mild bleeding, which includes nasal bleeding, gum bleeding, skin bleeding or petechiae, blood-stained sputum, bleeding hemorrhoids, or hematuria under a microscope, and did not require a change in the therapeutic plan; (2) major bleeding, which included digestive system bleeding, gross hematuria for 2 continuous days, hemoptysis, and required to change the therapeutic plan; and (3) life-threatening or severe bleeding, which might cause cardiac or respiratory arrest, or require surgical intervention, including intracranial hemorrhage, anemia (hematocrit < 0.2), and systolic blood pressure < 90 mm Hg.

### 2.4. Statistical Analysis

Data in this article were nonparametric except for the body weight and BMI. Statistical analysis was performed with SPSS (version 24). Measurement data were expressed as the mean ± standard deviation; comparisons between groups were expressed by independent two-sample *t*-test; categorical variables were expressed as a ratio and were assessed using the *χ*^2^ test; and the rank sum test was used to rank data. Comparisons of efficacy between two groups were analyzed by the Cox multivariate model. *P* < 0.05 was considered statistically significant.

## 3. Results

### 3.1. Patients

From January 2015 through December 2016, 138 patients were enrolled to this study: 86 received rivaroxaban treatment, and 42 were given LMWH and warfarin (standard therapy) with 10 patients changing regimen between the first and fifth months.  Table 1 summarizes the baseline demographics, treatment, and follow-up of patients in the current study. The mean age of patients in the rivaroxaban monotherapy group was higher than that in the standard therapy group. The ratio of intermediate-high-risk and PESI in the rivaroxaban monotherapy group was higher than that in the standard-therapy group. Other characteristics were not significantly different between the groups.

### 3.2. Clinical Outcome

As shown in Tables 2 and 3, the primary efficacy outcome was observed in 52 patients (40.48%) in the rivaroxaban group compared with 17 patients (60.47%) in the standard therapy group after 3 months (*P* = 0.033, new data *P* = 0.148). According to the Cox multivariate model, there was no significant difference in efficacy outcomes between the two groups after 3-month treatment (*P* = 0.798, 95% confidence interval (CI) 0.686 to 1.336) or more than 6-month treatment (*P* = 0.534, 95% CI 0.795 to 1.556). Moreover, thrombus absorption was not significantly different within 1 month between the two groups (*P* = 0.215). The rivaroxaban monotherapy group had a shorter hospitalization duration than that the standard therapy group, although this did not reach statistical significance. When the course of rivaroxaban monotherapy reached 6 months, we adjusted the dosage to 10 mg once daily, and no decline in efficacy was found compared with standard therapy. Rather, their conditions improved after treatment for 6 months.

The principal safety outcome is shown in Table 4. Severe bleeding episodes were not observed in any patients in the rivaroxaban group compared with 3 patients (7.14%) in the standard therapy group (*P* = 0.0132). Major bleeding was observed in one patient (1.16%) in the rivaroxaban group and two patients (4.76%) in the standard-therapy group (*P* = 0.043). However, there were two cases of major hemorrhage (one case with abdominal hemorrhage and another with serve hemoptysis) leading to death in the standard-therapy group. The rate of all-cause mortality was 3.49% in the rivaroxaban group and 9.52% in the standard-therapy group.

Table 5 shows that the primary efficacy outcome and principal safety outcome were similar between groups except for the rate of all-cause mortality in PE patients with cancer, which was higher than that of the nontumor group (61.11% vs 67.65%, *P* = 0.762).  Table 6 shows that efficacy outcome (*P* = 0.289) and safety outcome (*P* = 0.326) of rivaroxaban in patients with or without frailty were similar. For intermediate-high-risk patients, the efficacy of monotherapy with rivaroxaban is similar to thrombolysis treatment (absorption of thrombosis rate was 63.64% vs 46.15%, *P* = 0.392). And the incidence of bleeding was also similar in these 2 groups (minor bleeding 45.45% vs 76.92%, *P* = 0.245) (Table 7).

## 4. Discussion

Increasing numbers of phase III randomized clinical controlled trials have indicated that NOACs are not inferior to traditional standard therapy for the treatment of acute pulmonary embolism and have a better safety profile. Rivaroxaban was the first oral direct factor Xa inhibitor confirmed to be effective and safe in randomized controlled trials including EINSTEIN-DVT [9] and EINSTEIN-PE [10]. Westendrof et al. [11] evaluated the results of several real-world clinical studies including XALIA [12], XANTUS [13], GARFIELD [13, 14], and ORBIT-AF [15] and reaffirmed the safety and efficacy of rivaroxaban previously observed in RCTs.

In China, a series of clinical trials of rivaroxaban for the treatment of pulmonary embolism were also reported [16–19] after it was approved for the treatment of acute pulmonary embolism in 2017. The results of these studies also indicated that rivaroxaban may be used as an alternative treatment to standard therapy for the treatment of acute pulmonary embolism, with indiscriminate efficacy and lower bleeding risk. Because these studies were all clinical studies with small sample sizes and therefore provided limited data, the current study has added to the domestic clinical evidence regarding rivaroxaban for the treatment of PE.

The main results of our study demonstrated that rivaroxaban monotherapy is not inferior to standard therapy for the treatment of pulmonary embolism, consistent with our previous findings and those of the EINSTEIN-PE study. The current study found that the efficacy of rivaroxaban increased with time, suggesting it might be more suitable for patients who need long-term anticoagulation treatment. Overall, 4832 patients were enrolled in the EINSTEIN study, where the main efficacy outcome was the occurrence of recurrent thrombotic events. Our study provides more specific information regarding efficacy, which can be divided into four levels: disappearance, effective, no effect, and progressed to worse according to imaging findings.

Apart from enhanced long-term efficacy for the treatment of acute pulmonary embolism, rivaroxaban had better safety than standard therapy. These results are consistent with previous large-scale RCT studies. Regarding recurrence rates, major bleeding rates, and mortality rates, this study, which revealed the true clinical level, was closer to the real-world XALIA study compared with other RCT studies. However, the XALIA study had no data for pulmonary embolism. Therefore, the data in the current study should provide support for clinical decision-making. The analysis of subgroups in acute pulmonary embolism was specific to this study and showed that rivaroxaban was comparable to standard treatment and LMWH for the treatment of frail patients, cancer patients, and high-risk patients, confirming evidence for its clinical application.

To ease the economic burden of patients, the dose of rivaroxaban was reduced to 10 mg once daily for patients in the rivaroxaban monotherapy group who still need anticoagulation therapy for at least 3 months, and no decrease in efficacy was observed. There was a significant difference in efficacy between the two groups at 6 months, which suggested that for Chinese patients, after 3 months of treatment with 20 mg once daily, a reduced dose of 10 mg once daily can be effective.

This was a semiretrospective and semiprospective study and therefore had some limitations common to retrospective studies: (1) bias of patient memory, because the patients were diagnosed with PE during 3 years, many patients were already cured, and the degree of memory regarding bleeding events might be inaccurate; (2) bias of data integrity, most patients lacked records of CTPA or examination of urine at a specific timepoint; and (3) bias of loss of follow-up, the rate of lost to follow-up for the warfarin and rivaroxaban groups was 2.38% and 8.13%, respectively. This study also included hospital admission bias, namely, patients hospitalized in the First Affiliated Hospital of Guangzhou Medical University do not fully represent the overall population of patients with acute pulmonary embolism treated in other hospitals.

In the subgroup analyses (frail patients, cancer patients, or intermediate-high risk patients) of this study, the number of cases was not large enough, and it will be necessary to conduct large-scale research to verify the results of this study. Furthermore, because few patients with tumors were injected with LMWH subcutaneously in the clinic, we could not compare rivaroxaban monotherapy with LMWH therapy in tumor patients; therefore, it will be necessary to collect more LMWH therapy cases to conduct further research.

## 5. Conclusions

The efficacy of the rivaroxaban regimen was not inferior to the standard therapy. With an extension of treatment time, the rivaroxaban monotherapy group had a better efficacy and lower incidence of bleeding episodes compared with the standard-therapy group. The efficacy of rivaroxaban did not decrease when its dose was reduced to 10 mg once daily.

## Figures and Tables

**Table 1:** Demographic and clinical data of patients.

Variable	Group	*N*	Rivaroxaban	Warfarin	*χ* ^2^/*T*	*P* value
Sex	F	67	45 (52.33%)	22 (52.38%)	0.000	0.995
	M	61	41 (47.67%)	20 (47.62%)		

Combined with DVT	Without	69	48 (55.81%)	21 (50%)	0.384	0.536
	With	59	38 (44.19%)	21 (50%)		

HAS-BLED	Lower danger	122	85 (98.84%)	37 (88.1%)	7.288	0.007∗∗
	Higher danger	6	1 (1.16%)	5 (11.9%)		

Risk stratification	Lower danger	27	22 (25.58%)	5 (11.9%)	0.384	0.536
	Intermediate-lower danger	60	46 (53.49%)	14 (33.33%)		
	Intermediate-high danger	36	15 (17.44%)	21 (50%)		
	Higher danger	5	3 (3.49%)	2 (4.76%)		

Expected course of treatment	3 months	53	34 (39.53%)	19 (45.24%)	0.378	0.539
	6 months	75	52 (60.47%)	23 (54.76%)		

Thrombolysis	Without	107	75 (87.21%)	32 (76.19%)	2.498	0.114
	With	21	11 (12.79%)	10 (23.81%)		

Age	—	128	58.08 ± 14.52	52.55 ± 14.80	2.012	0.046∗

Weight	—	125^a^	64.84 ± 12.09	61.76 ± 12.06	1.347	0.180

BMI	—	125^a^	24.28 ± 3.61	23.22 ± 3.37	1.583	0.116

PESI	—	128	82.15 ± 27.06	71.83 ± 24.34	2.091	0.039∗

sPESI	—	128	1.02 ± 1.10	0.88 ± 0.99	0.711	0.478

^a^Missing number is 3.

**Table 2:** Efficacy outcomes.

Variable	Group	*N*	Rivaroxaban (86)	Warfarin (42)	*χ* ^2^	*P* value
Reexamination after 1 month	Disappeared	49	37 (43.02%)	12 (28.57%)	1.5396	0.2147
	Effective	46	30 (34.88%)	16 (38.1%)	0.0810	0.7760
	No effect or progressed	10	6 (6.98%)	4 (9.52%)	0.2343	0.6283
	Reappear	0	0 (0%)	0 (0%)		
	—	23	13 (15.12%)	10 (23.81%)		

Reexamination after 3 months	Disappeared	69	52 (60.47%)	17 (40.48%)	2.0916	0.1481
	Effective	34	22 (25.58%)	12 (28.57%)	0.0950	0.7579
	No effect or progressed	12	7 (8.14%)	5 (11.9%)	0.4267	0.5136
	Reappear	0	0 (0%)	0 (0%)		
	—	7	5 (5.81%)	8 (19.05%)	0.0571	0.8111

**Table 3:** Efficacy in patients receiving the expected course of treatment.

Duration	Efficacy	*N*	Rivaroxaban (52)	Warfarin (23)	*χ* ^2^	*P* value
1 month	Disappeared	27	22 (42.31%)	5 (21.74%)	1.8740	0.1710
	Effective	30	20 (38.46%)	10 (43.48%)	0.1003	0.7514
	No effect or progressed	6	4 (7.69%)	2 (8.7%)	0.0201	0.8874
	—	12	6 (11.54%)	6 (26.09%)		

3 months	Disappeared	38	30 (57.69%)	8 (34.78%)	1.6519	0.1987
	Effective	19	13 (25%)	6 (26.09%)	0.0074	0.9313
	No effect or progressed	9	6 (11.54%)	3 (13.04%)	0.0301	0.8623
	—	9	3 (5.77%)	6 (26.09%)		

6 months	Disappeared	39	31 (59.62%)	8 (34.78%)	1.8911	0.1691
	Effective	19	11 (21.15%)	8 (34.78%)	1.1692	0.2796
	No effect or progressed	7	5 (9.62%)	2 (8.7%)	0.0145	0.9043
	—	10	5 (9.62%)	5 (21.74%)		

**Table 4:** Safety of patients with expected course of treatment.

Variable	Group	*N*	Rivaroxaban (86)	Warfarin (42)	*χ* ^2^	*P* value
Reexamination after 1 month	No bleeding	55	40 (46.51%)	15 (35.71%)	0.7656	0.3816
	Mild bleeding	54	36 (41.86%)	18 (42.86%)	0.0066	0.9350
	Serve bleeding	3	0 (0%)	3 (7.14%)	6.1429	0.0132
	Major bleeding	2	0 (0%)	2 (4.76%)	4.0952	0.0430
	—	14	10 (11.63%)	4 (9.52%)		

Reexamination after 3 months	No bleeding	49	38 (44.19%)	11 (26.19%)	2.3872	0.1223
	Mild bleeding	61	40 (46.51%)	21 (50%)	0.0721	0.7884
	Serve bleeding	3	0 (0%)	3 (7.14%)	6.1429	0.0132
	Major bleeding	2	0 (0%)	2 (4.76%)	4.0952	0.0430
	—	13	8 (9.3%)	5 (11.9%)		

Reexamination after 6 months	No bleeding	46	37 (43.02%)	9 (21.43%)	3.6617	0.0557
	Mild bleeding	65	41 (47.67%)	24 (57.14%)	0.4982	0.4803
	Serve bleeding	4	1 (1.16%)	3 (7.14%)	3.2292	0.0723
	Major bleeding	3	1 (1.16%)	2 (4.76%)	1.5596	0.2117
	—	10	6 (6.98%)	4 (9.52%)		

**Table 5:** Efficacy and safety of rivaroxaban in patients with pulmonary embolism combined with or without tumor.

Duration	Efficacy	*N*	PE with tumor (18)	PE without tumor (68)	*χ* ^2^	*P* value
Efficacy	Disappeared	57	11 (61.11%)	46 (67.65%)	0.0917	0.7620
	Effective	16	2 (11.11%)	14 (20.59%)	0.6871	0.4072
	No effect	5	1 (5.56%)	4 (5.88%)	0.0026	0.9592
	Progressed	2	1 (5.56%)	1 (1.47%)	1.0212	0.3122
	Reappeared	2	0 (0%)	2 (2.94%)	0.5294	0.4669
	—	4	3 (16.67%)	1 (1.47%)		

Bleeding	No bleeding	42	9 (50%)	33 (48.53%)	0.0063	0.9367
	Mild bleeding	38	7 (38.89%)	31 (45.59%)	0.1446	0.7038
	Serve bleeding	1	1 (5.56%)	0 (0%)	3.7778	0.0519
	Major bleeding	1	0 (0%)	1 (1.47%)	0.2647	0.6069
	—	4	1 (5.56%)	3 (4.41%)		
	Death	3	3 (16.67%)	0 (0%)	11.333	<0.001

**Table 6:** Efficacy and safety of rivaroxaban in patients with PE combined with or without frailty.

	*N*	PE with frailty (22)	PE without frailty *n* = 64	*χ* ^2^	*P* value
Efficacy				-0.72	0.471
Disappeared	55	12 (54.55%)	43 (67.19%)	1.35	0.289
Effective	20	8 (36.36%)	12 (18.75%)	2.846	0.092
No effect	5	1 (4.55%)	4 (6.25%)	<0.001	1
Progressed	2	0 (0.00%)	2 (3.13%)		0.552

Bleeding				-1.363	0.173
Bleeding	39	8 (36.36%)	31 (48.44%)	0.963	0.326
Mild bleeding	27	13 (59.09%)	27 (42.19%)	1.88	0.170
Serve bleeding	1	0 (0.00%)	1 (1.56%)		0.744
Major bleeding	1	1 (4.55%)	0 (0.00%)		0.256

**Table 7:** Comparison of rivaroxaban and thrombolytic therapeutic in intermediate-high-risk patients.

		Thrombolysis (13)	Rivaroxaban (11)	*χ* ^2^	*P* value
Efficacy				-1.479	0.139
	Disappeared	6 (46.15%)	7 (63.64)	0.734	0.392
	Effective	6 (46.15%)	2 (18.18)	1.028	0.311
	No effect	0 (0.00%)	2 (18.18)		0.199
	Progressed	0 (0.00%)	0 (0.00)		
	Reappeared	1 (7.69%)	0 (0.00)		0.542

Bleeding				2.517	0.113
	Bleeding	3 (23.08%)	5 (45.45%)	0.524	0.496
	Mild bleeding	10 (76.92%)	5 (45.45%)	1.354	0.245
	Serve bleeding	0 (0.00%)	0 (0.00%)		
	Major bleeding	0 (0.00%)	0 (0.00%)		
	Death	0 (0.00%)	2 (18.18%)		0.199

## Data Availability

The data used to support the findings of this study are available from the corresponding author upon request.

## Abbreviations

APE: Acute pulmonary embolism
BMI: Body mass index
CTPA: Computed tomographic pulmonary angiography
INR: International normalized ratio
LMWH: Low-molecular-weight heparin
NOACs: Nonvitamin K-dependent new oral anticoagulants
NVAF: Nonvalvular atrial fibrillation
RCT: Randomized controlled trial.
